# Skincare Benefits of a Postbiotic Ferment Produced Through Djon Djon Mushroom Fermentation by *Saccharomyces*

**DOI:** 10.1111/jocd.70067

**Published:** 2025-02-19

**Authors:** Chunhong Pu, Doudou Shi, Michael Ingrassia, Harvey Gedeon, Tye Chu, Jiachan Zhang, Changtao Wang

**Affiliations:** ^1^ Beijing Key Lab of Plant Resource Research and Development Beijing Technology and Business University Beijing P. R. China; ^2^ Institute of Cosmetic Regulatory Science Beijing Technology and Business University Beijing P. R. China; ^3^ Beishang Jiamei (Beijing) Technology Co. Ltd. Beijing P. R. China; ^4^ Dermegen Inc. Hauppauge New York USA; ^5^ Laboratoire de Furcy LLC El Portal Florida USA

**Keywords:** Djon Djon, fermentation, inflammation, oxidative stress, photodamage, RNA‐sequencing

## Abstract

**Background:**

Djon Djon is a particularly special black mushroom indigenous to Haiti that has a long history in both their cuisine and in traditional medicine. Centuries of folkloric utilization of theses “medicinal” botanicals tend to indicate the presence of a potentially efficacious western medicine entity.

**Objectives:**

With the advantages afforded by both traditional medicines and fermentation, we endeavored to investigate if fermentation of Djon Djon mushrooms can provide skin care benefits.

**Methods:**

In this study, active Djon Djon fermentation broth (DDF) was obtained using *Saccharomyces*, and anti‐inflammatory efficacy was assessed in cultured systems using human keratinocytes and fibroblasts, exposed to either UVB or H_2_O_2_ respectively. In addition, RNA‐Seq technology was employed to further characterize the mechanisms of DDF following ultraviolet irradiation.

**Results:**

Characterization of the DDF displayed a high number of polysaccharides and peptides present following fermentation, that function to scavenge intracellular ROS, decrease MDA content, while increasing the levels of CAT, COL‐I, and HA in HSF induced by H_2_O_2_. In addition, levels of pro‐inflammatory factors (IL‐6, IL‐1β, and TNF‐α) were decreased in UVB irradiated HaCaT cells that had been treated with DDF. Analysis of cellular RNA indicated that DDF altered the DEGs involved in the AGE‐RAGE signaling pathway suggesting that this signaling cascade is inhibited by DDF. Additionally, DDF also influenced the metabolism of arachidonic acid, histidine, and phenylalanine, which are involved in inflammatory processes.

**Conclusion:**

DDF can alleviate oxidative stress damage caused by hydrogen peroxide and photodamage caused by UVB, and the mechanism by which DDF protects skin cells is revealed, displaying the potential benefits of fermented DjonDjon in skincare.

## 1 Introduction

Djon Djon is the Kreyòl word for fungus, referring to a kind of black mushroom found in the mountains in northern Haiti. It can be picked from the crannies and crevices of dead wood during the rainy season, which lasts from August to October. Djon Djon belongs to *Psathyrella*, and is rich in protein, polysaccharides, ash, minerals, vitamins, and prebiotics [1, 2]. In the United States, most markets that serve Haitian communities import these dried mushrooms. They typically sell for around $64 a pound, making them the most expensive ingredient in Haitian cooking.

Mushrooms have played a role in food and medicine for centuries, even before their benefits were investigated and identified. Generally, mushrooms are a class of macro‐fungi used in cooking and possess high nutraceutical and medicinal properties [3, 4]. Some of the reported benefits include anti‐diabetes, antioxidation, antivirus, antibacterial, bone protection, kidney protection, and liver protection, [5, 6, 7]. Mushrooms contain a rich source of polysaccharides, proteins, polysaccharide‐protein complexes, polyphenols, ergosterols, and vitamins [8, 9]. More recently, the use of mushroom extracts has been reported to help improve the skin [10, 11, 12]. Mushroom extract containing cosmeceuticals have been reported to have strong clinical functionality, including anti‐inflammatory, anti‐tyrosinase, antioxidant, antibacterial, UV‐protective and skin soothing properties [13, 14, 15]. Furthermore, reported safety data on mushroom extracts indicate favorable compatibility in skin care products [16, 17].

Human skin is directly exposed to ultraviolet radiation from the sun, and as such, the need for therapies to protect the skin is of great importance. Consequently, the presence of UV‐protective factors in mushrooms has drawn researcher's attention, as oxidative and inflammatory damage has been suggested to play important roles in the pathogenesis of skin photoaging [18].

UV is classified into UVA (320–400 nm), UVB (280–320 nm), and UVC (100–280 nm). The atmosphere filters out most of the solar UVB and all UVC. Thus, UV that reaches the earth surface is a combination of UVA and UVB. The longer wavelengths of UVA allows for deeper skin penetrability and as a result, it is UVA that impacts dermal fibroblasts, affecting both elastic fibers and collagen residing in the dermis. UVB's shorter, high energetic wavelengths has less penetrability and consequently affects epidermal keratinocytes. It is here that the high energy of UVB is absorbed by DNA resulting in TT dimers and 6,4 photoproducts. It has been established that chronic and intense UVB exposure causes photoaging, while acute UVB exposure can cause sunburn which can eventually lead to photocarcinogenesis [19, 20, 21].

One of the significant consequences of UV exposure is the generation of reactive oxygen species (ROS). ROS are potent molecular agents inflicting oxidative injury to essential skin constituents, including proteins, lipids, and DNA, thus perpetuating the aging phenomenon [22, 23]. Many publications have proved that decreasing the production of ROS while promoting its clearance are important mechanisms for cell survival and prevention of cellular senescence [24, 25, 26, 27]. H_2_O_2_ is a major inducer of reactive oxygen and is often responsible for causing oxidative damage in Human Skin Fibroblasts (HSF). Extracts from mushrooms have been proven to exhibit ROS scavenging activity [15, 28, 29, 30]. In addition, another approach of preventing UV damage is to reduce inflammation [31, 32, 33]. Interleukin‐1β (IL‐1β), lnterleukin‐6 (IL‐6) and tumor necrosis factor‐α (TNF‐α) are common biomarkers that coincide with inflammation. Although there is limited information of Djon Djon mushroom in the literature, reports exist about the anti‐inflammatory effects of other mushrooms, such as *Boletus aereus* polysaccharides [34], lentinan [35], hydroalcoholic extract from *Agaricus blazei* Murill [36], *Grifola frondosa* [37], etc.

The skin‐protective biological activities of *Saccharomyces* ferments has recently been recognized. *Saccharomyces cerevisiae* fermented sugarcane straw displayed excellent radical scavenging (ABTS) activity as well as inhibiting elastase and tyrosinase enzymes [38]. Furthermore, *Passiflora edulis* Sims Peel fermented by *Saccharomyces cerevisiae* was reported to enhance human epidermal cell defense against UVB damage [39]. Both in vitro, and in vivo data have been reported. Interestingly, a fermented combination of *Saccharomyces* and *Lactobacillus* displayed potent alleviation of sensitive skin syndrome [40]. Each of these mushrooms provide an opportunity to synergize with *Saccharomyces* in the development of fermented skincare products.

Based on this, *Saccharomyces* was used in the study to ferment Djon Djon mushroom. The Djon Djon fermentation broth (DDF) was collected and used in our evaluations. We employed the use of human skin keratinocytes (HaCaT) and human skin fibroblasts (HSF) in the ultraviolet radiation experiments. In further studies using HSF, an H_2_O_2_‐induced oxidative stress model was established to investigate the potential antioxidant effect of our DDF ferment. To evaluate the effect of DDF on the production of inflammatory mediators, a UVB‐induced HaCaT cell photodamage model was established. Furthermore, molecular biology was employed and the expression of genes were evaluated in order to study the molecular mechanisms of DDF inhibition of inflammation. Gene Ontology (GO) and Kyoto Encyclopedia of Genes and Genomes (KEGG) pathway enrichment analysis were performed to find out the key biological changes that were involved in these processes. Additionally, protein interactions were performed in the study.

## 2 Materials and Methods

### 2.1 Materials

*Psathyrella Ciorinceps* (Djon Djon Mushroom) was obtained from Huida Biopharmaceutical Co. Ltd. *Saccharomyces* was obtained from China General Microbiological Culture Collection Center (CGMCC). Human skin fibroblast (HSF) and HaCaT were both purchased from the Cell Resource Center at the Institute of Basic Medicine, Chinese Academy of Medical Sciences. Dulbecco's Modified Eagle Medium (DMEM), fibroblast medium, fetal bovine serum (FBS), phosphate buffer saline (PBS), penicillin, streptomycin, 0.25% trypsin (with EDTA), and 1% antibiotic‐antimycotic solution (100 U/mL penicillin/streptomycin and 100 U/mL amphotericin) were acquired from Thermo Fisher Scientific. (Carlsbad, CA, USA); Cell lysis buffer (Western blotting and IP), phenylmethanesulfonyl fluoride, ROS, CAT, MDA and BCA Protein Assay kits were purchased from Beyotime Biotechnology Co. Ltd. EasyScript OneStep gDNA Removal and cDNA Synthesis SuperMix reverse transcription kit, TransStart Top Green qPCR SuperMix kit were purchased from Beijing TransGen Biotech Co. Ltd. Cell Counting kits‐8 were purchased from Beijing Biorigin Biotechnology Co. Ltd. COL‐I Assay kit was purchased from Cloud‐Clone Corp.

All other chemicals were of analytical grade or complied with the standards required for cell culture experiments.

### 2.2 Preparation of Djon Djon Fermentation Broth (DDF)

The *Saccharomyces* strain utilized for fermentation was maintained in yeast extract peptone dextrose (YPD) media or on YPD agar plates. Seed cultures were initially transferred to 500‐mL Erlenmeyer flasks containing 1% Djon Djon Mushroom dried powder at a dose of 5% inoculation and grown for 2 days at 30°C with shaking at 200 rpm. Fermentation broth (DDF) was then centrifuged (5000 rpm, 30 min), collected and then sterilized (121°C, 15 min).

### 2.3 Phytochemical Analysis

#### 2.3.1 Total Phenols

The content of total phenols was quantified using the Folin–Ciocalteu Reagent [41]. The concentration of phenol was calculated using a Gallic acid standard curve (*y* = 6.2947*x* + 0.01; *y* is the absorbance, and *x* is the concentration of Gallic Acid in Mg/mL; *R* ^2^ = 0.9986), with the sample solvent used as the blank control.

#### 2.3.2 Total Flavonoids

The total flavonoids content was quantified by the dowd method similar to that described by Jia Zhishen et al. [42]. The concentration of flavonoids was calculated from a standard curve comprised of Rutin (*y* = 0.8796*x* + 0.0037; *y* is the absorbance and, *x* is the concentration of Rutin in Mg/mL; *R* ^2^ = 0.9902).

#### 2.3.3 Total Polysaccharides

The content of total polysaccharides was quantified using the phenol‐sulfuric acid method [43]. Total polysaccharides were calculated from a standard curve of glucose (*y* = 6.9611*x* + 0.0081; *y* is the absorbance and, *x* is the concentration of glucose in Mg/mL; *R* ^2^ = 0.9996).

#### 2.3.4 Reducing Sugar

Reducing sugar content was determined using the dinitrosalicylic acid (DNS) method [44]. The equation of the standard curve for reducing sugar was as follows: *y* = 0.9116*x* − 0.0289 (*R* ^2^ = 0.9996), where, *y* is the absorbance and x is the concentration of reducing sugar in mg/mL.

#### 2.3.5 Peptides

Peptides were quantified using the Biuret method [45]. Briefly peptides are incubated under alkaline conditions in the presence of Copper. Following the formation of a purplish‐red complex, it can be analyzed spectrophotometrically at a wavelength of 540 nm. The standard curve was obtained as follows: *y* = 0.0013*x* + 0.0128 (*R* ^2^ = 0.9935); *y* is the absorbance and *x* is the concentration of peptides in mg/mL.

### 2.4 Cell Culture and Model Establishment

HSF and HaCaT cells were both grown in a DMEM medium supplemented with 10% (HaCaT) or 15% (HSF) FBS and 1% Penicillin–Streptomycin Solution, at 37°C and 5% CO_2_. Typically, HSF used in these experiments were between 5 and 10 passages.

#### 2.4.1 The Establishment of an H_2_O_2_ ‐Induced HSF Oxidative Stress Model and Cytotoxicity

HSF were seeded into a 96‐well plate at a density of 8 × 10^3^ cells/well. After 12 h incubation, the cells were exposed to different concentrations of H_2_O_2_ for 2 h. Cell viabilities were measured using CCK‐8 (Cell Counting Kit 8, Thermo Fisher Scientific Instruments Ltd. China). The half maximal inhibitory concentration (IC_50_) values for H_2_O_2_‐induced HSF oxidative stress were selected to establish the model. Similarly, cells viabilities by different concentrations of DDF for 24 h were also measured using the CCK‐8 assay to obtain the working concentration of DDF.

In the study, the IC_50_ was reached following incubation of HSF cells for 2 h with 1000 μM H_2_O_2_. Based on the concentration data corresponding to 80% cell viability (CV_80_), the following experiment was performed with 1% DDF. Detailed information was exhibited in the Supporting Information.

#### 2.4.2 The Establishment of a UVB‐Induced HaCaT Photodamage Model and Cytotoxicity

HaCaT cells were seeded into 96‐well plates at a density of 1 × 10^5^ cells/well. Cell viability was assessed under various treatment conditions, including multiple concentrations of DDF (0%–40%) and UVB exposure levels (0–30 mJ/cm^2^ at 310 nm) (UV07‐II, Ningbo, China), respectively. Cell number was determined using the CCK8 assay kit as described above. The IC_50_ dose of UVB was determined to be 10 mJ/cm^2^ (data not shown).

### 2.5 Hydroxyl Radical Scavenging Activity

Hydroxyl radical scavenging activity was measured following previous studies [46]. DDF samples ranging from 1.56% to 50% were evaluated, and hydroxyl radical scavenging activity (%) of DDF was calculated using the formula below.

Hydroxyl radical scavenging activity (%) = [(A_0_ − A_i_ + A_j_)/ A_0_] × 100%.

Where, A_0_ is the absorbance of DDF replaced by distilled water; A_i_ is the absorbance of DDF, and A_j_ is the absorbance of H_2_O_2_ replaced by distilled water.

### 2.6 Determination of Cellular ROS, CAT, MDA and COL‐I

HSF were cultured into a 6‐well plate at a density of 3 × 10^5^ cells/well. Upon reaching approximately 80% cell density, the HSF cells are subjected to stimulation with H_2_O_2_, followed by a triple wash process using PBS. Subsequently, the sample is promptly introduced and incubated for a duration of 24 h. Levels of ROS, catalases (CAT), malondialdehyde (MDA) and collagen type I (COL‐I) were all quantified following manufacturer's instructions and data was normalized to protein content. Ascorbic acid (50 μg/mL) was used as the positive control in these studies, and. total protein contentwas determined using a BCA total protein assay kit.

### 2.7 Determination of IL‐6, IL‐1β and TNF‐α

HaCaT cells were cultured into a 6‐well plate and at a density of 5.0 × 10^5^ cells/well. When the HaCaT cell density reached 80%, cells were washed 3 times with PBS prior to irradiation with UVB. Immediately following irradiation, cells were treated with DDF for 24 h. Levels of secreted IL‐6, IL‐1β and TNF‐α were determined following the manufacturer's instructions. Dipotassium glycyrrhizinate (DG) (1%) was used as the positive control in these studies.

### 2.8 Transcriptome Sequencing

In order to gain a better understanding on the mechanisms responsible for the effects of DDF following UVB irradiation, molecular biological methods were employed, using RNA Seq technology. Following UVB irradiation (10 mJ/cm^2^), DDF treated HaCaT cells were collected and total RNA was extracted using TRIzol Reagent according the manufacturer's instructions (Invitrogen).

RNA purity was assessed following removal of genomic DNA with DNase I (TaKara). RNA quality was determined using a 2100 Bioanalyzer (Agilent) and quantified spectrophotometrically using a ND‐2000 spectrophotometer (NanoDrop Technologies). Only high‐quality RNA samples (OD260/280 = 1.8 ~ 2.2, OD260/230 ≥ 2.0, RIN ≥ 6.5, 28S:18S ≥ 1.0, > 1 μg) was used to construct sequencing library. RNA‐seq transcriptome library was prepared using a TruSeqTM RNA sample preparation Kit from Illumina (San Diego, CA) using 1 μg of total RNA. In brief, the procedures used followed the literature [24].

### 2.9 Screening for Differentially Expressed Genes (DEGs)

Detailed information including platform, quality control, bioinformatics tools, and the RNA sequencing data analysis are all exhibited in the Table S1.

Data were analyzed on the free online platform of Majorbio Cloud Platform (www.majorbio.com) [47], with statistical analysis of DEGs conducted using the DESeq2 package. Statistical significance (*p*‐value) and the fold change (FC) for each gene were calculated to denote its expression difference between libraries.

Statistical significance for DEGs between the Control and the Model group were determined having a *p*‐value < 0.05 and a |log2FC| > 1. With respect to DEGs between the DDF and the Model group, statistical significance was determined as having a *p*‐value < 0.01 and a |log2FC| > 4. The significant Gene Ontology (GO) terms for biological process (PB), molecular function (MF), and cellular component (CC) of DEGs were enriched using the *p* < 0.05. In addition, the significantly enriched pathways were identified from the Kyoto Encyclopedia of Genes and Genomes (KEGG) database using the *p* < 0.05 as the criterion.

### 2.10 Protein–Protein Interaction (PPI) Network and Topological Analysis

PPI network analysis was performed using the STRING database (https://cn.string‐db.org/). The STRING database systematically collects and integrates protein–protein interactions‐physical interactions and functional associations, and contains proteomic information on human proteins [48].

One hundred twenty‐three DEGs between the DDF and the Model group were integrated with the PPI network to obtain weighed PPI networks. All seven active prediction methods (Textmining, Experiments, Databases Co‐expression, Neighborhood, Gene Fusion Co‐occurrence) were used in the analysis, with a minimum required interaction score of medium confidence (0.400) and the species restricted to “ *Homo Sapiens* ”. Visualization of the interaction network was performed using Cytoscape version 3.10.0. The topological analysis of these hub nodes was calculated using the CytoNCA plugin. The algorithm of betweenness centrality was used to rank the nodes and obtain key targets for visual analysis.

### 2.11 Statistical Analysis

All experiments were performed using at least triplicate samples, and data were expressed as mean ± standard deviation (SD). The data was analyzed using GraphPad Prism 9.0 (GraphPad Software, USA) software. Data were analyzed using a one‐way analysis of variance (ANOVA) and significant means (*n* = 3) were tested using the Dunnett's multiple comparisons test (*p < 0.05; **p < 0.01, n.s. *p* > 0.05).

## 3 Results

### 3.1 Phytochemicals in DDF and Their Antioxidant Capacities In Vitro

In our first study, we set out to quantitate the levels of total polysaccharides, protein and other active components in DDF. Polysaccharides levels within the ferment were quantified to be at the highest concentration at 4.720 ± 0.1659 mg/mL, and peptides were quantified at 3.7560 ± 0.0326 mg/mL, while flavonoids were the least abundant (Table 1). Mushroom polysaccharides have a tremendous history in the food, pharmaceutical, and cosmetics industries due to their inherent biological benefits, including antioxidant, anti‐inflammatory, anti‐tumor, antibacterial, hypoglycemic, blood pressure lowering, radiation resistance, immune regulation, and others [49, 50]. Natural active peptides such as myostatin [51] and glutathione [52] are highly sought after in the food, pharmaceutical and cosmetic industries, and peptides in mushrooms have displayed potential skincare effects, as well as other health benefits [53, 54]. The significant levels of polysaccharides and peptides in DDF suggests that it may possess great functionality.

**TABLE 1:** Phytochemical characterization of DDF. Values represent data mean ± SD (*n* = 3).

Items	Content (mg/mL)
Total phenols	0.096 ± 0.0025
Total flavonoids	0.009 ± 0.0017
Total polysaccharides	4.720 ± 0.1659
Reducing sugar	0.041 ± 0.0006
Peptides	3.757 ± 0.0326

**FIGURE 1:**
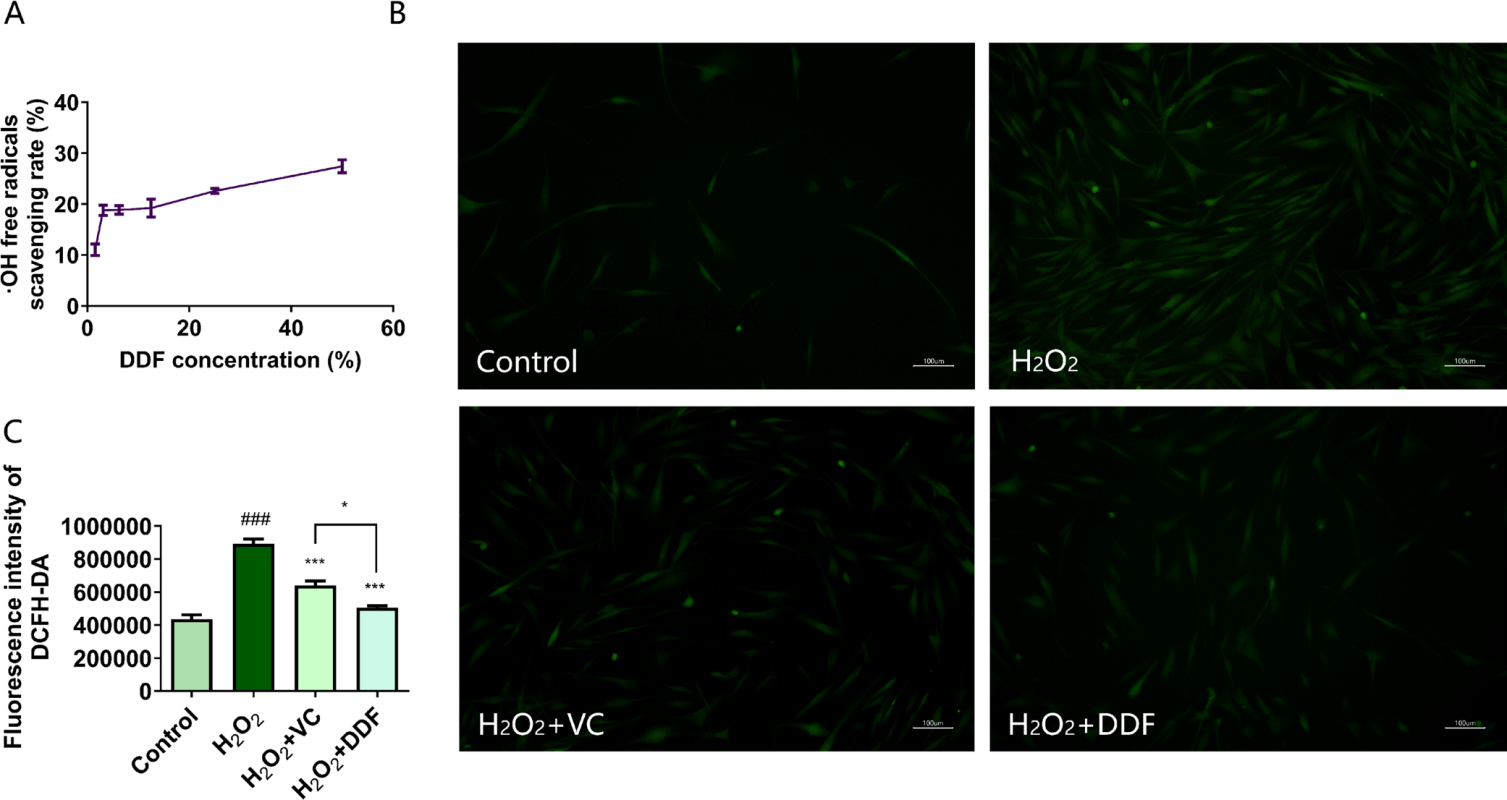
Antioxidant functionality of DDF. (A) Scavenging rates of hydroxyl radicals (•OH) using a biochemical assay. DDF ranging from 1.56% to 50% was evaluated. (B, C) showed the effects of DDF (1%) and Ascorbic acid (VC, Positive control, 50 μg/mL) on ROS production induced by H_2_O_2_. (B) Representative fluorescence photos of different treatments on HSFs. (C) Fluorescence intensities. Control represents cells with no treatment. The group named H_2_O_2_ represents cells stressed with 1000 μM H_2_O_2_ for 2 h. Results expressed as the mean ± SD (*n* = 3). Statistical significance was determined by ANOVA analysis. ###, *p* < 0.001, compared to the Control. *, *p* < 0.05, ***, *p* < 0.001, compared to H_2_O_2_.

The toxicity of DDF to HSF and HaCaT cells was assessed. As illustrated in Figure S2, cell viability exhibited a dose‐dependent relationship with DDF concentration, with no cytotoxicity observed at concentrations below 1.25%. Experimentation of the antioxidant capacity of DDF was evaluated in two separate studies (Figure 1). In one study evaluating DDF's ability to scavenge hydroxy radicals, dose‐dependent inhibition was observed using 1.56% to 50% of DDF (Figure 1A). In a second study, the intracellular levels of ROS were measured as illustrated in Figure 1B,C. Hydrogen peroxide robustly induced levels of intracellular reactive oxygen species (ROS) known to cause damage to cellular DNA and initiate apoptosis. Fluorescence imagery depicting intracellular ROS levels in HSF were captured using inverted fluorescence microscopy (Figure 1B). The fluorescence intensity is directly proportional to the level of intracellular ROS. Upon exposure to H_2_O_2_, there was a significant increase in the ROS fluorescence intensity within HSF cells; however, treatment with DDF and VC resulted in a noticeable decrease in the ROS fluorescence intensity in HSF cells (Figure 1C) with 1% DDF proving to be more effective at scavenging ROS than 50 μg/mL ascorbic acid in this study.

### 3.2 DDF Mitigation of H_2_O_2_ Induced Oxidative Stress on CAT, MDA, COL I and HA

Hydrogen peroxide and the hydroxyl radical are the two major ROS known to be produced by aerobic metabolism in organisms [55]. The excessive ROS can activate lipid peroxidation and increase malondialdehyde (MDA) levels, which cause cell metabolism disorders [56, 57]. Enzymatic detoxification of H_2_O_2_ is performed mainly by CAT [57].

**FIGURE 2:**
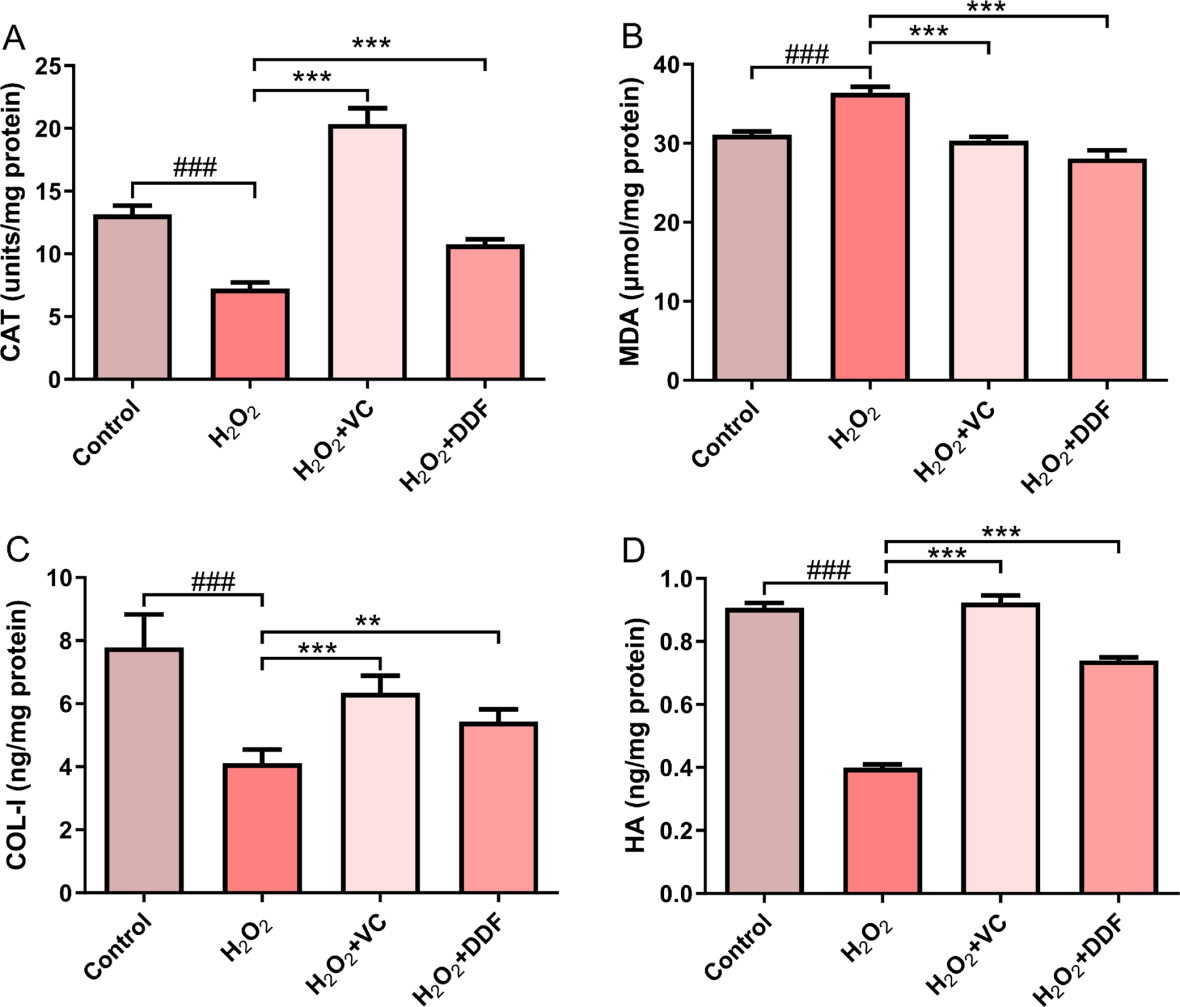
Effects of DDF (1%, H_2_O_2_ + DDF group) on: (A) CAT, (B) MDA, (C) COL‐I and (D) HA production. The group named H_2_O_2_ represents cells stressed by 1000 μM H_2_O_2_ for 2 h. VC (50 μg/mL, H_2_O_2_ + VC group) was used as the positive control. Statistical significance was determined by ANOVA analysis. ###, *p* < 0.001, compared to the Control. **, *p* < 0.01, ***, *p* < 0.001, compared to the H_2_O_2_.

In these studies, the influence of DDF on CAT and MDA levels were evaluated. The presence of H_2_O_2_ significantly reduced the amount of intracellular CAT, while the co‐addition of DDF significantly increase the amount of CAT (Figure 2A, *p* < 0.001). Similarly, H_2_O_2_ addition significantly increased MDA levels in HSF (Figure 2B, *p* < 0.001). However, the co‐addition of DDF significantly reduced the increase in MDA levels induced by H_2_O_2_, below that of the control group (Figure 2B, *p* < 0.001).

Collagen type I (COL‐I) and hyaluronic acid (HA) are the major extracellular matrix components (ECM) of the skin [58]. The addition of DDF significantly enhanced the levels of COL‐I and HA in HSF cells (Figure 2C,D), which were reduced by the negative effects of H_2_O_2_.

### 3.3 DDF Inhibits Cytokine Markers of Inflammation Following UVB Exposure

**FIGURE 3:**
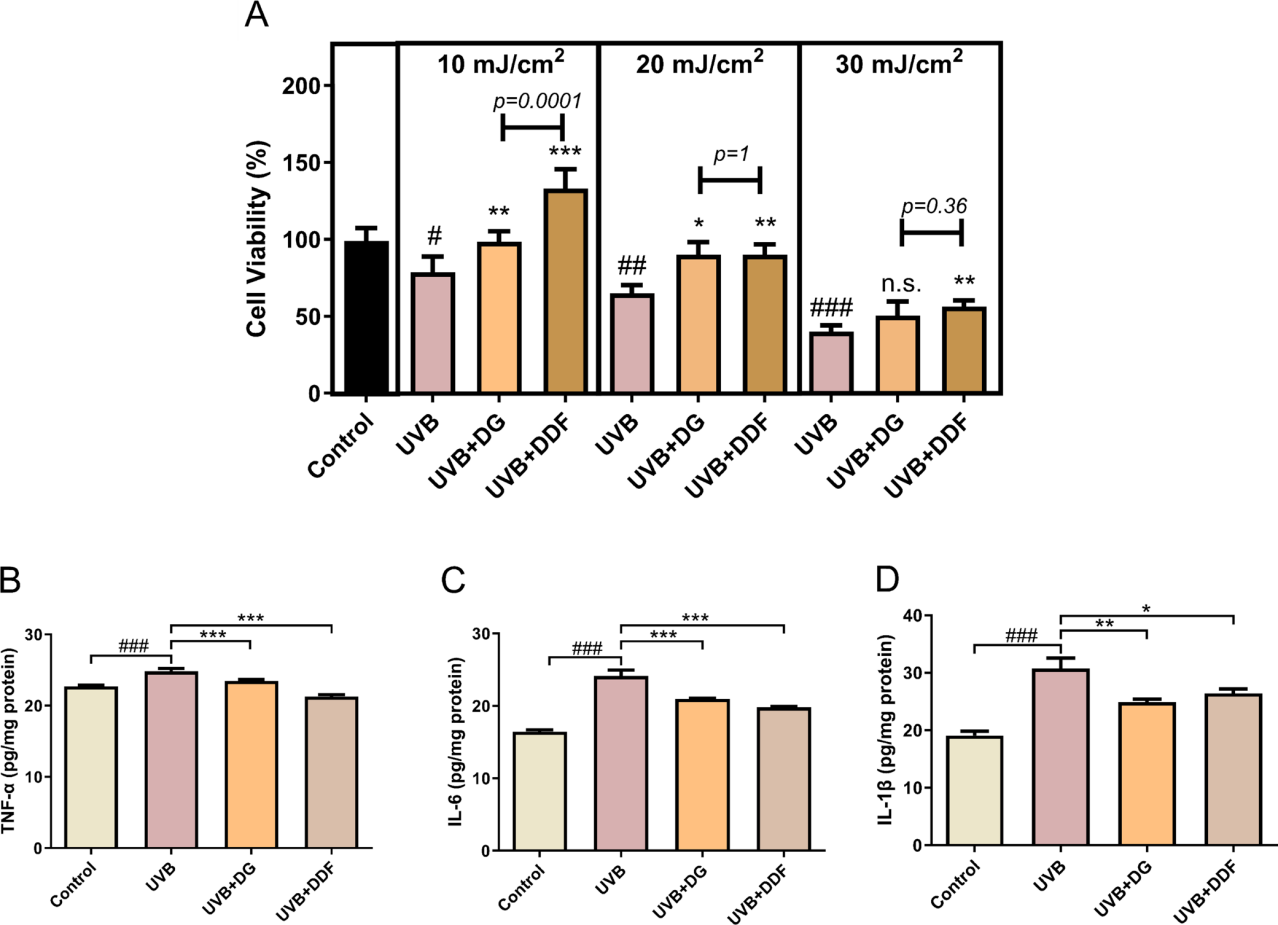
Cell viability and reduction of inflammation HaCaT cells incubated with DDF. Cell viability was evaluated in cells exposed to three different intensities of UVB (10, 20 and 30 mJ/cm^2^). Cytokine levels of TNF‐α (B), IL‐6 (C) and IL‐1β (D) were measured under 10 mJ/cm^2^ of UVB. 1% Dipotassium glycyrrhizinate (DG, UVB + DG group) was used as the positive control. Statistical significance was determined by ANOVA analysis. #, *p* < 0.05, ##, *p* < 0.01, ###, *p* < 0.001, compared with the Control. *, *p* < 0.05, **, *p* < 0.01, ***, *p* < 0.001 compared with the UVB group.

The photodamage will vary with the intensity of UVB. The direct results obtained were the cell viabilities and the proinflammatory factors secreted. In this study, different intensities of UVB were employed to stimulate inflammatory cytokine secretion from HaCaT cells. Cell viability was assessed to help in selecting the appropriate intensity of UVB, and as expected, significant decreases in cell viability was observed with 10, 20 and 30 mJ/cm^2^ (Figure 3A). Both DDF and dipotassium glycerrizhinate (DG) can significantly protect against UVB induced reduction in cell viability. More importantly, DDF displayed a better protective effect than DG (the positive control) at 10 mJ/cm^2^ UVB irradiation. Doses of 20 and 30 mJ/cm^2^ seemingly do more harm to HaCaT cells than that of 10 mJ/cm^2^, thus making it more difficult to overcome the cell damage induced at these higher intensities. Interestingly, there were no significance between DDF and DG at these greater intensities (20 and 30 mJ/cm^2^). Due to these results, 10 mJ/cm^2^ was chosen to assess cellular inflammatory responses to UVB.

Proinflammatory cytokines, TNF‐α, IL‐6 and IL‐1β, were measured using 10 mJ/cm^2^ UVB. Significant increases of all these cytokines were observed following UVB irradiation (Figure 3B–D). Consistent with the cell viability results (Figure 3A), both DDF and DG inhibited the secretion of the proinflammatory factors from UVB irradiated HaCaT cells.

### 3.4 GO and KEGG Analysis of DDF in the HaCaT Cell UVB Photodamage Model

A total of 1576 genes were identified in HaCaT cells subjected to the UVB‐induced photodamage (the Model group) relative to normal HaCaT cells (the Control group), of which 729 were upregulated and 847 were downregulated, using *p* < 0.05 and |log_2_FC| > 1 as the thresholds. A total of 618 up‐regulated and 852 down‐regulated DEGs were screened in the DDF treated group relative to the Model group, using the criteria of *p* < 0.01 and |log_2_FC| > 4.

**FIGURE 4:**
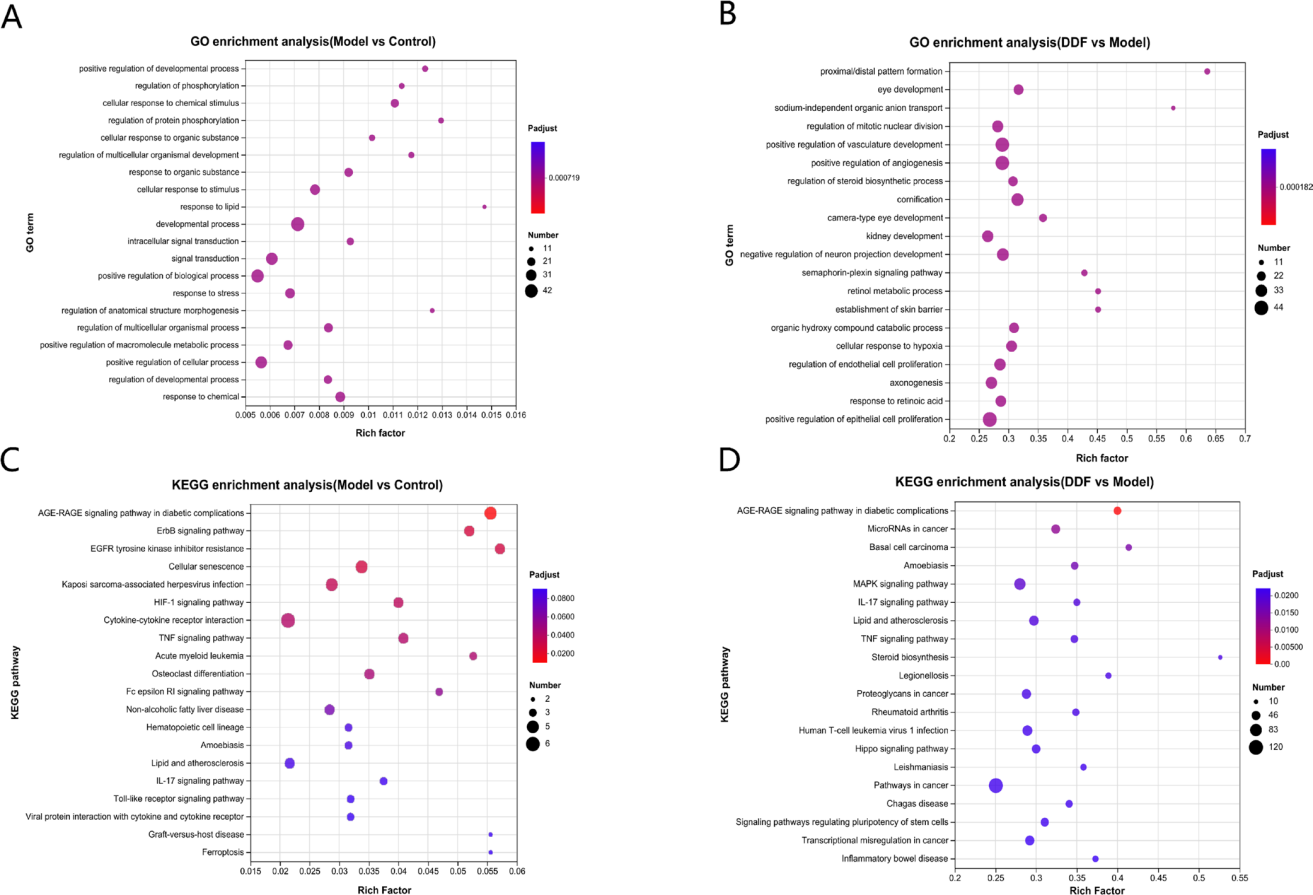
Top 20 GO terms (A, B) and KEGG pathways (C, D) of DEGs enriched in RNA‐sequencing. (A) and (C), DEGs selected from the group between Model (UVB) and Control; (B, D), DEGs selected from the group between DDF and Model. The Rich factor representsthe ratio of the number of target genes divided by the number of all the genes in each GO term or KEGG pathway. The pool size indicates the gene counts, and the color reflects the value of *Padjust*.

A GO enrichment analysis of these DEGs was performed using the online bioinformatics platform of Majorbio Cloud Platform (www.majorbio.com) [47]. Top 20 GO biological processes (Figure 4A,B) and KEGG pathway biological processes (Figure 4C,D) of DEGs between different groups (Model vs. Control, and DDF vs. Model) were exhibited separately in Figure 4.

The top 5 altered biological processes (BP) by UVB exposure were, positive regulation of developmental process, regulation of phosphorylation, cellular response to chemical stimulus, regulation of protein phosphorylation, and cellular response to organic substance (Figure 4A). Among the top 20 GO terms enriched by the DEGs of DDF versus Model group, DEGs were significantly involved in the terms, such as sodium‐independent organic anion transport, positive regulation of angiogenesis, establishment of skin barrier, cellular response to hypoxia, and regulation of endothelial cell proliferation (Figure 4B).

KEGG pathway enrichment analysis displayed that DEGs encoding the AGE‐RAGE signaling pathway, ErbB signaling pathway, cellular senescence, cytokine/cytokine receptor interaction, and TNF signaling pathway etc. were significantly regulated following UVB treatment. These pathways are all reported to be involved in inflammation and photoaging [59], consistent with the effects of UVB irradiation.

Notably, AGE‐RAGE binding could activate many signaling pathways related to inflammation, oxidative stress, and apoptosis. RAGE activation has been demonstrated to induce NF‐κB, which in turn increase the expression of proinflammatory cytokines and activate the MAPK signaling pathway, thus leading to inflammation, proliferation, and apoptosis [60, 61]. Dysregulation of the TNF and ErbB signaling pathway can also mediate inflammation via the MAPK signaling pathway [62, 63]. Furthermore, IL‐17 signaling pathway and Toll‐like receptor signaling pathway were also included in top 20 terms but with *Padjust* values both above 0.05 (Figure 4C). In our studies, DDF treatment affected the genes involved in pathways, such as AGE‐RAGE signaling pathway, MAPK signaling pathway, IL‐17 signaling pathway, and TNF signaling pathway, with *Padjust* values all below 0.02 (Figure 4D), thereby, promoting the suppression of inflammation.

### 3.5 Analysis of the DEGs Changed due to DDF

**FIGURE 5:**
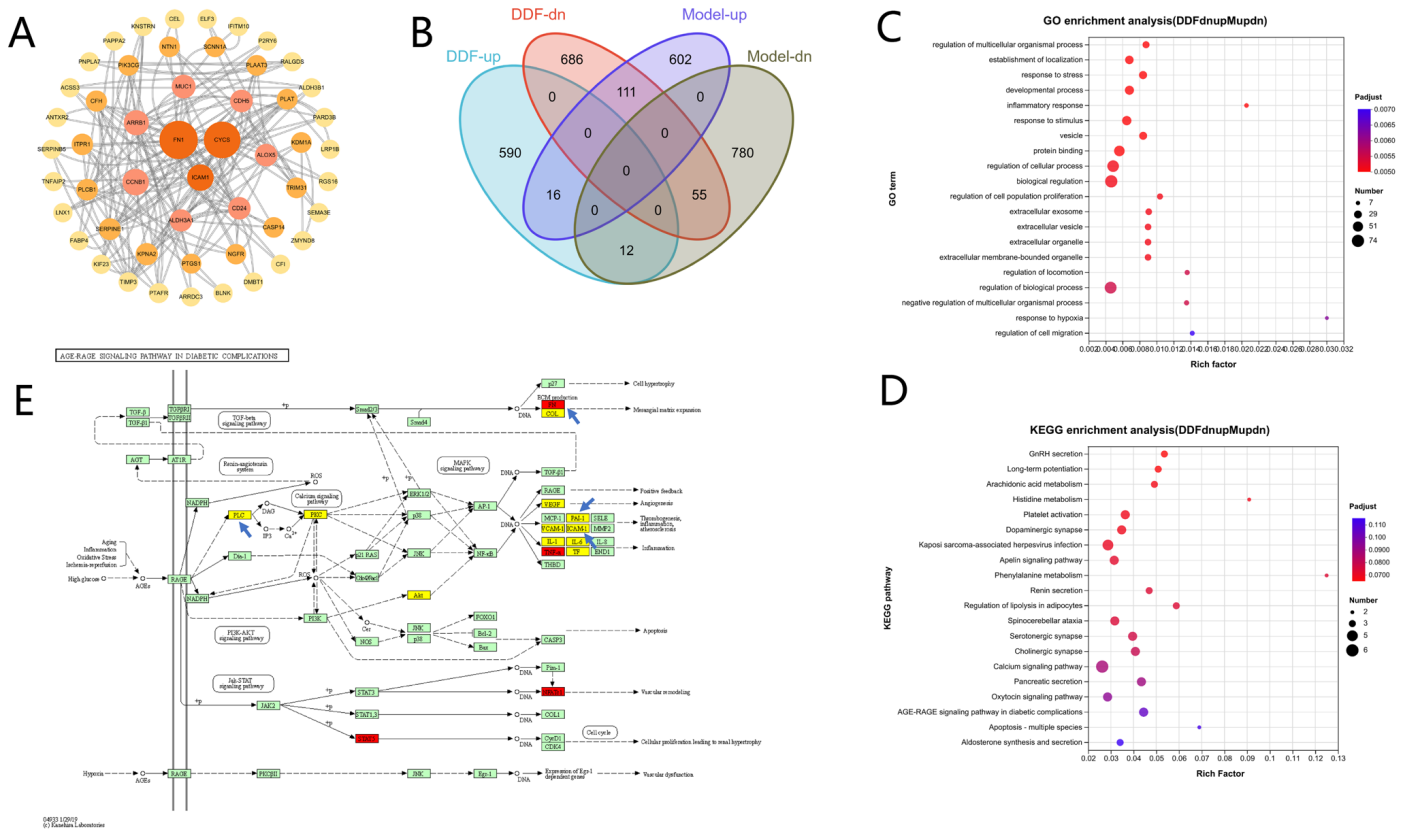
(A) Network of protein–protein interactions with a common goal, with darker colors indicating greater importance. Nodes represent target proteins and lines represent interrelationships. Informatic analysis displaying DDF reversal of DEGs affected by UV (Model) Irradiation. (B) Venn diagram. DDF‐up and DDF‐dn represented the up‐ and down‐regulated DEGs selected from the group between DDF and Model. Model‐up and Model‐dn represented the up‐ and down‐regulated DEGs selected from the group between Model and Control. The up‐regulated 111 DEGs in Model‐up were found suppressed by DDF. The inhibited 12 DEGs in Model‐dn were induced by DDF. The two overlapping areas (DDFdnupMupdn) were chosen for further GO and KEGG enrichment analysis. The top 20 GO terms and KEGG pathways were exhibited in (C) and (D). The Rich factor, represents the ratio of the number of target genes divided by the number of all the genes in each GO term or KEGG pathway. The size of pot indicated the gene counts, and the color reflected the value of *Padjust*. (E) AGE‐RAGE signaling pathway. Red represents up‐regulated DEGs by DDF. Yellow represents down‐regulated DEGs by DDF. The four genes marked with the blue arrows belong to the group DDFdnupMupdn.

The Venn diagram in Figure 5B showed the overlap of Model and DDF DEGs. A total of 123 up‐ or down‐regulated DEGs changed to the opposite regulated ones. Among the 123 DEGs (DDFdnupMupdn), a great proportion of UVB upregulated genes (111 DEGs) were inhibited by DDF (Figure 5B). GO enrichment analysis showed that DDF altered the genes involved in the response to stress, response to stimulus, inflammatory response, regulation of cell population proliferation etc. (Figure 5C).

Metabolically, KEGG pathway analysis indicated several pathways that have been influenced by cells incubated with DDF, including arachidonic acid metabolism, histidine metabolism, and phenylalanine metabolism (Figure 5D). In some cases, arachidonic acid released from membrane phospholipids can be further metabolized to prostaglandins (PGs) in response to inflammatory stimuli. Among the prostanoid family, prostaglandin E2 (PGE2) is an inflammatory regulator that is involved in cellular differentiation, inflammation and cancer [64]. The reduction in PGE2 synthesis is one of the molecular mechanisms of some nonsteroidal anti‐inflammatory drugs [65]. Leukotreines, another class of notable pro‐inflammatory mediators downstream of arachidonic acid, has been influenced by DDF as well. Conversely, some of the regulated genes, specifically those of the resolvin family of anti‐inflammatories, specifically lipoxins, function by means of stimulating efferocytosis [66], granulocyte apoptosis [67], modulating endothelial activation and reducing leukocyte recruitment [68].

Among the 123 DEGs reversed by DDF, there were four genes participating in the AGE‐RAGE signaling pathway, including ICAM1, SERPINE1, FN1, and PLCB1, displayed in Figure 5E (blue arrow). Apart from those, there were an additional 14 genes affected by DDF involving the AGE‐RAGE signaling pathway, such as the suppressed AKT3, PRKCA, COL3A1, COL4A3, COL4A4, IL‐1, and IL‐6 (yellow nodes in Figure 5E), as well as the up‐regulated TNF, STAT5A, and NFATC1 (red nodes in Figure 5E).

### 3.6 PPI Network Analysis

In this study, we overlapped our gene list (123 nodes) with the PPI network and removed the noninteracting edges. After topological analysis, the resulting network consisted of 52 nodes with 160 edges, as shown in Figure 5A.

The PPI network was analyzed based on BC values, and the top 10 nodes were identified as FN1, CYCS, ICAM1, CCNB1, ARRB1, MUC1, CDH5, ALOX5, CD24, and ALDH3A. After screening with BC > 40 and DC ranging from 4 to 30, the top 26 nodes are shown in Table II (in descending order of betweenness centrality).

## 4 Discussion

There are many mechanisms that have been reported to play a role in skin damage induced by UV. One such mechanism involves UV light stimulation of excessive ROS which subsequently induce oxidative stress. Molecules that possess the ability to scavenge free radicals have been observed to provide ample protection against oxidative stress damage. The levels of intracellular ROS were significantly decreased after treatment with DDF, and levels of ROS were significantly lower than the positive control (VC, *p* = 0.02). Furthermore, we evaluated the accumulation of lipid peroxidation product MDA and observed a reduction following incubation with DDF. Lastly, the cellular antioxidant enzyme CAT was found in high level after DDF treatment, further displaying the antioxidant capacity of DDF.

Another process involved in UV induced skin damage is the onset of inflammation. UV radiation usually causes acute phase responses and stimulates inflammatory factors in the skin. These factors include the interleukins, such as IL‐1, IL‐6, and IL‐8, monocyte chemoattractant protein (MCP)‐1, TNF‐α, and granulocyte‐macrophage colony‐stimulating factor (GM‐CSF), which collectively play crucial roles in various inflammatory problems such as atopic dermatitis and psoriasis [69, 70, 71]. UVB is a major risk factor for inflammation. In this study, a UVB induced HaCaT model exhibited high levels of IL‐6, IL‐1β, and TNF‐α, in agreement with the reported literature [69, 72].

Today's wholistic movement has driven people to seek natural active ingredients from plants or microorganisms to address many ailments, including protection against skin inflammatory damage. The anti‐inflammatory effects of mushroom‐derived active compounds have been widely reported to exhibit anti‐tumor, immune‐enhancing, hypolipidemic, hypoglycemic, and antimicrobial properties [73, 74, 75, 76]. In vivo studies mainly relate to liver, brain, kidney, blood, and intestine [77, 78], however there are not many studies addressing the skin. Nevertheless, mushroom's pleiotropic systemic benefits lays the theoretical foundation for its possible dermatological benefits.

The beneficial effects of yeast‐related products in the field of skincare have been documented. The natural compound β‐Glucan are polysaccharides found in cell walls of yeast, and fungi (including mushrooms). The skin health benefits of β‐glucan include, antioxidant activity, anti‐wrinkle activity, anti‐UV, wound healing, moisturization and skin permeation absorption, have all reported and reviewed by Du et al. [79]. Peptides from *Saccharomyces cerevisiae* showed no cytotoxic effects on HaCaT and HDF cells and exerted positive effects on the production of collagen, hyaluronic acid, fibronectin, and elastin [80]. Lee et al. produced the Bioconversion Oji complex using a two‐step bio conversion process including macadamia seed oil and typical plants as the substrates for *Candida bombicola*. The results showed that the complex had skin improving efficacies (skin anti‐inflammatory, moisturizing, and barrier improvement) both in vitro and in clinical studies [81].

In this study, DDF was produced by *Saccharomyces* with Djon Djon freeze‐dried powder as the major substrate. It is clear that the ingredients of DDF were derived both from the mushroom and from the microorganisms, and the efficacy of this fermentation complex affords has been verified in these studies. Peroxide (H_2_O_2_) induced HSF oxidative stress model and a UVB‐induced HaCaT photodamage model were both created, to ascertain the antioxidation and anti‐inflammation aspects of DDF.

Further studies were employed using RNA‐Seq technology to ascertain the anti‐photodamage mechanisms of DDF. Among the top 10 signaling pathways returned by KEGG pathway enrichment analysis, DDF could alter the genes expression associated with AGE‐RAGE, MAPK, TNF, and IL‐17 signaling pathways, which collectively regulates diverse biological processes, such as inflammation, proliferation, host defense, and vascular remodeling. Specifically, four of 18 DEGs (ICAM1, SERPINE1, FN1, and PLCB1) (Figure 5E) were modulated by DDF in the AGE‐RAGE pathway, a less‐characterized circuit that activates inflammatory and profibrogenic signaling [82]. The generation of free radicals and the expression of SERPINE1 can be inhibited by CAT [83]. Under the influence of DDF, CAT levels in cells were elevated, and transcriptome sequencing confirmed that SERPINE1 expression was indeed suppressed. The expression of downstream genes IL‐1(IL‐1 alpha and IL‐1beta) and IL‐6 were suppressed after DDF treatment, which were consistent with the ELISA results (Figure 3C,D). Interestingly, the expression of TNFα in RNA‐Seq differed when compared with the ELISA results (Figure 3B). This may be due to the differences in transcription and translation levels of TNFα. VCAM1(vascular cell adhesion molecule 1) and ICAM1(intercellular adhesion molecule 1) belonging to the immunoglobulin superfamily, were both downregulated by DDF. Potential expression of VCAM1 and ICAM1 on endothelial cells could increase leukocyte adherence and migration [84], which are necessary for efficient immune response against invading stimuli on one hand. While, on the other hand, these processes may also result in tissue damage and promote chronic inflammatory states [85]. The production of ROS can promote the expression of ICAM1 and VCAM1 [86]. DDF could inhibit the production of ROS, which was consistent with the results of RNA sequencing, and the expression of ICAM1 and VCAM1 was also inhibited. Our data suggest that DDF may play a role in suppressing the damage caused by inflammation.

Angiogenesis and inflammation are two closely linked hallmarks in many disease processes [87]. Excessive vascularization is beneficial to the transport of inflammatory cells and acute permeability of the neo‐formed vessels promotes the development of edema, especially in cancer [88]. DDF exhibited anti‐inflammatory activity, partly due to the down‐regulation of angiogenesis, exemplified by the decrease of VEGFA (vascular endothelial growth factor A) gene expression according to RNA‐Seq (Figure 5E).

Fibronectin is a broadly expressed component and a major adhesive molecule of the extracellular matrix (ECM). Notably, DDF in the study up‐regulated the expression of FN1(fibronectin 1), which plays a role in a wide range of cellular processes, such as regulating cell‐matrix adhesion and diffusion, cell migration and cytoskeleton by interacting with the integrin receptor family [89, 90]. Another ECM component, collagen type 1 (COL‐I), was detected at a high level after the addition of DDF, seemingly implying a recovery of HSF following photodamage. In fact, the production of FN1 is regulated by HA, and the increase of HA can promote the expression of FN1, which is consistent with the results of this study [91]. Although different cell models were used in these studies, the effect of DDF was effective on the repair of damaged cells in all models tested.

Cytochrome c(CYCS) is well known as the penultimate electron transport protein of the eukaryotic respiratory chain [92]. Its main function is to act as an electron carrier in the mitochondrial respiratory chain, transferring electrons from complex III to complex IV. It is capable of acting in cytogenesis and proliferation, and plays an antioxidant role in the production and elimination of oxygen and hydrogen peroxide [93, 94]. The antioxidant effects of DDF, as observed in the study by increasing CAT content and decreasing MDA content, may be closely linked to the expression of CYCS, which was identified as one of the top 2 nodes in the PPI network. Although different cell models were used in this study, the effect of DDF on repairing damaged cells was positive and mutually confirmed.

In addition, DDF altered the gene expressions involved in arachidonic acid metabolism, histidine metabolism, and phenylalanine metabolism, which some metabolites such as PGE2, histidine, and phenylalanine, act as major mediators of inflammatory processes [64, 95, 96].

Future investigations, validating and evaluating the expression and regulation of some key genes, will shed light on related signaling pathways and gene interactions, offering a more comprehensive understanding of the fungal ferment' mechanism against H_2_O_2_ and UVB damage. An integrated analysis on multiple data sets may also be included to identify more key targets. These efforts aim to determine the mechanisms behind the anti‐photodamage effect of DDF, and promote the application in both health and skincare products. Additionally, the study explores the beneficial effects of fermentation products after microbial action on the skin, and we will further elucidate the role of microorganisms in this process in the future.

## 5 Conclusion

In these studies, a unique mushroom, Djon Djon, was fermented by *Saccharomyces*. The broth (DDF) was collected, and phytochemicals were analyzed to characterize the beneficial factors it contained. Based on the results, we found that DDF could scavenge the intracellular ROS levels, decrease MDA content, and increase the levels of CAT, COL‐I, and HA in HSF stressed by H_2_O_2_. A UVB‐induced HaCaT photodamage model was used to evaluate the anti‐inflammatory capacity of DDF. The pro‐inflammatory factors (IL‐6, IL‐1β, and TNF‐α) stimulated in our model system were attenuated after DDF treatment. RNA‐Seq technology was employed to further elucidate the mechanisms of DDF on photodamage. DDF altered the DEGs involved in the AGE‐RAGE signaling pathway indicating at least one possible mechanism responsible for the anti‐photodamage effect of DDF. Lastly, DDF also influenced the metabolism of arachidonic acid, histidine, and phenylalanine, all key players in inflammatory processes. We hope these studies provide a theoretical basis for DDF application in skincare.

## Author Contributions

Jiachan Zhang, Chunhong Pu and Changtao Wang planned the study. Jiachan Zhang, Chunhong Pu and Michael Ingrassia performed and analyzed experiments. Doudou Shi and Jiachan Zhang helped with the RNA‐Seq and statistical analysis. Jiachan Zhang, Chunhong Pu, Doudou Shi and Harvey Gedeon wrote the initial manuscript. Tye Chu contributed to grammar editing and data analysis. All the authors provided intellectual contribution.

## Ethics Statement

This study did not include any animal or human tests and thus would not require an Ethics Statement.

## Conflicts of Interest

The authors declare no conflicts of interest.

## Supporting information


Data S1.

## Data Availability

The data that support the findings of this study are available from the corresponding author upon reasonable request.
