# Supplementary material for: Skincare Benefits of a Postbiotic Ferment Produced Through Djon Djon Mushroom Fermentation by Saccharomyces
Source: J Cosmet Dermatol. 2025 Feb 19;24(2):e70067. doi: 10.1111/jocd.70067 (PMC11836922; doi:10.1111/jocd.70067)
Supplement: Supplementary file 1 — Data S1. [file JOCD-24-e70067-s001.docx]

**Supplementary Material**

**Figure S1. The establishment of H_2_O_2_-induced oxidative damage model.**


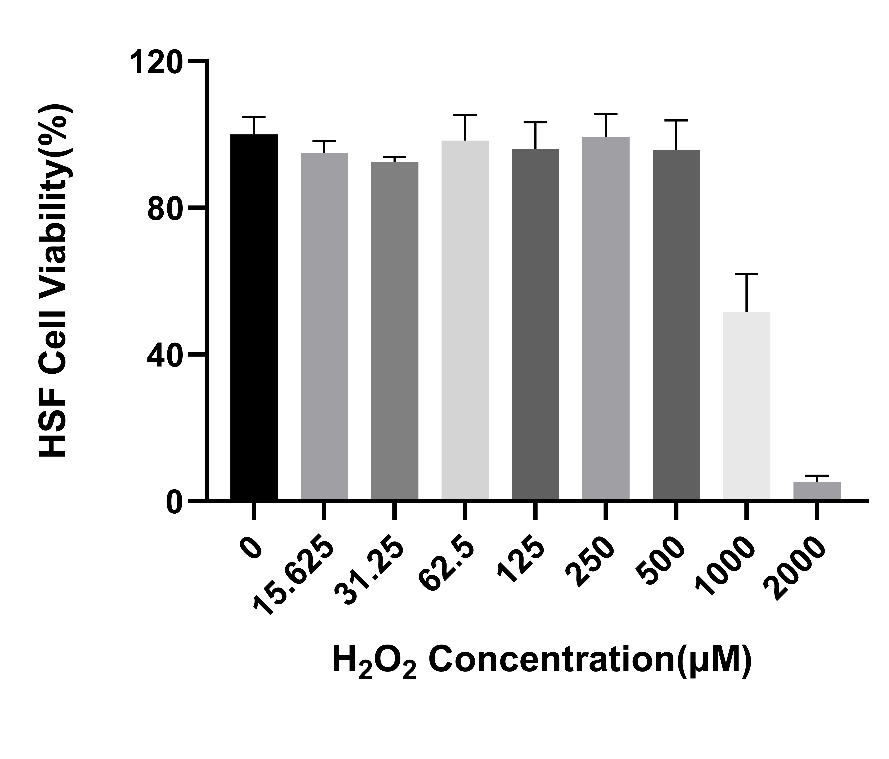


**Figure S2. The cell viability of DDF on HSF and HacaT cells.**

**
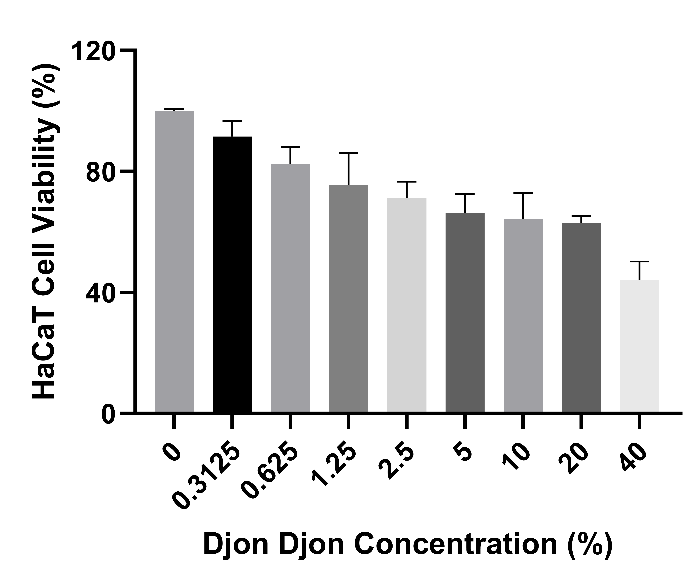

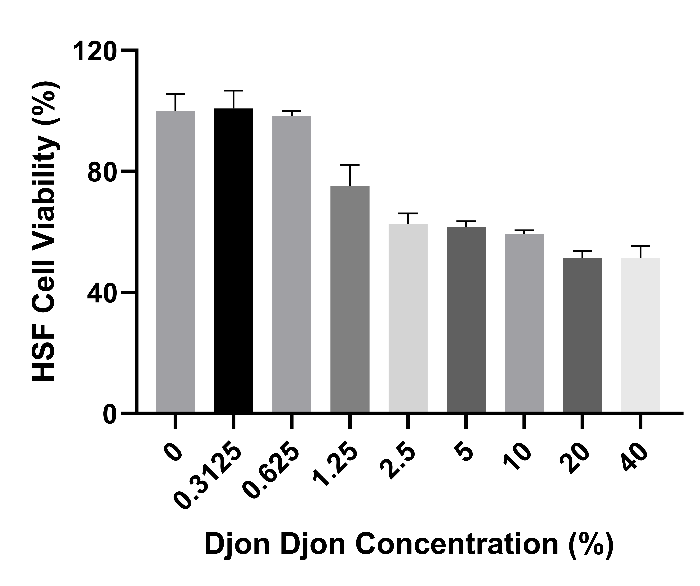
**

***The critical information like platform, quality control, bioinformatics tools and the RNA sequencing data analysis (quality control, assembly, and mapping) were exhibited in the following parts.***

**Library preparation and Sequencing**

RNA-seq transcriptome library construction and sequencing were performed using the TruSeq^TM^ RNA sample preparation kit according to the manufacturer’s instructions (Illumina, San Diego, CA, USA) at Shanghai Majorbio Bio-pharm Biotechnology Co., Ltd. (Shanghai, China). Firstly, poly(A) mRNA was purified according to the polyA selection method by oligo(dT) beads and then fragmented by fragmentation buffer. Secondly, the SuperScript double-stranded cDNA synthesis kit (Invitrogen, CA, USA) served to synthesize double-stranded cDNA with random hexamer primers (Illumina). Then the synthesized cDNA was subjected to end-repair, phosphorylation and ‘A’ base addition according to Illumina’s library construction protocol. Libraries were size selected for cDNA target fragments of 300 bp on 2% Low Range Ultra Agarose followed by PCR amplified using Phusion DNA polymerase (New England Biolabs, Boston, MA) for 15 PCR cycles. After being quantified by TBS380 fluorometer (Turner Biosystems, USA), the paired-end RNA-seq sequencing library was sequenced utilizing the NovaSeq Xplus sequencer (2 × 150bp read length).

**Quality control Read mapping**

The raw paired end reads were trimmed and quality controlled by fastp^[1]^ with with default parameters. Then clean reads were separately aligned to reference genome with orientation mode using HISAT2software^[2]^. The mapped reads of each sample were assembled by StringTie^[3]^ in a reference-based approach.

**Gene Expression Analysis**

The data were analyzed on the free online platform of Majorbio Cloud Platform ([www.majorbio.com](http://www.majorbio.com))^[4]^. Briefly, to identify the differentially expressed genes (DEGs) between the different samples, the expression level of each transcript was calculated according to transcripts per million reads (TPM) method. RSEM^[5]^ (http://deweylab.biostat.wisc.edu/rsem/) was used to quantify gene abundances. Statistical analysis of DEGs was conducted using the DESeq2 package. A *P* value and the fold change (FC) for each gene were calculated to denote its expression difference between libraries.

**RNA Sequencing Data Analysis: Quality Control, Assembly, and Mapping**

There was a total of 72.52 Gb Clean Data obtained in RNA-Seq analysis. The Clean Data of each sample reached more than 6.29 Gb and the percentage of Q30 bases was more than 94.7%.

The clean reads were mapped to the reference genome (Genome assembly: GRCh38.p13, http://asia.ensembl.org/Homo_sapiens/Info/Index) and the detailed mapping output is summarized in the following Table S1.

**Table S1.** Summary of trimming and read mapping results of the sequences generated from different samples.

| Sample | Raw reads | Clean reads | Total reads | Q30(%) | Total mapped | Multiple mapped | Uniquely mapped |
| --- | --- | --- | --- | --- | --- | --- | --- |
| Control1 | 58223530 | 57664918 | 57664918 | 95.24 | 55876272(96.9%) | 1795402(3.11%) | 54080870(93.78%) |
| Control2 | 49411134 | 48909162 | 48909162 | 95.19 | 47462141(97.04%) | 1754727(3.59%) | 45707414(93.45%) |
| Control3 | 47057114 | 46504632 | 46504632 | 94.7 | 45077969(96.93%) | 1574312(3.39%) | 43503657(93.55%) |
| Model1 | 43025064 | 42638948 | 42638948 | 95.19 | 41337832(96.95%) | 1336741(3.14%) | 40001091(93.81%) |
| Model2 | 64981138 | 64314460 | 64314460 | 95.05 | 62420957(97.06%) | 2118805(3.29%) | 60302152(93.76%) |
| Model3 | 49530646 | 49019312 | 49019312 | 95.13 | 47518633(96.94%) | 1538228(3.14%) | 45980405(93.8%) |
| DDF1 | 62074532 | 61499248 | 61499248 | 95.95 | 59587991(96.89%) | 1792888(2.92%) | 57795103(93.98%) |
| DDF2 | 67812152 | 67217132 | 67217132 | 96.17 | 65193618(96.99%) | 2028186(3.02%) | 63165432(93.97%) |
| DDF3 | 52594338 | 52138992 | 52138992 | 96.17 | 50594068(97.04%) | 1499126(2.88%) | 49094942(94.16%) |

**Note**: Three replicates of Control (Control-1, -2 and -3), Model (Model-1, -2 and -3) and DDF (DDF-1, -2 and -3) treatments were carried out in RNA-seq analysis.

**Reference**

[1] Chen S , Zhou Y , Chen Y , et al. fastp : an ultra-fast all-in-one FASTQ preprocessor. 2018.

[2] Kim D, Langmead B, Salzberg S L. HISAT: a fast spliced aligner with low memory requirements[J].Nature methods, 2015, 12(4): 357-360.

[3] Pertea M, Pertea G M, Antonescu C M, et al. StringTie enables improved reconstruction of a transcriptome from RNA-seq reads[J]. Nature biotechnology, 2015, 33(3): 290-295

[4]Ren, Yi, Yu, Guo, Shi, Caiping, Liu, Linmeng, Guo, Quan, Han, Chang, Zhang, Dan, et al. 2022. Majorbio Cloud: A One-Stop, Comprehensive Bioinformatic Platform for Multiomics Analyses. iMeta 1, e12.

[5] Li B, Dewey CN. RSEM: accurate transcript quantification from RNA-Seq data with or without a reference genome. BMC Bioinformatics 2011; 12:323.
